# Supplementary material for: Polyelectrolyte elastomer-based ionotronic sensors with multi-mode sensing capabilities via multi-material 3D printing
Source: Nat Commun. 2023 Aug 10;14:4853. doi: 10.1038/s41467-023-40583-5 (PMC10415297; doi:10.1038/s41467-023-40583-5)
Supplement: Supplementary file 1 — Supplementary Information [file 41467_2023_40583_MOESM1_ESM.pdf]

Supplementary Information

Polyelectrolyte elastomer-based ionotronic sensors with multi-mode sensing capabilities via multi-material 3D printing

Caicong Li[1,2,#], Jianxiang Cheng[1,3,#], Yunfeng He[1,2,#], Xiangnan He[1,3], Ziyi Xu[1,2], Qi Ge[1,3,*], Canhui Yang[1,2,*]

[1]Shenzhen Key Laboratory of Soft Mechanics & Smart Manufacturing, Southern University of Science and Technology, Shenzhen, Guangdong, 518055, P.R. China

[2]Soft Mechanics Lab, Department of Mechanics and Aerospace Engineering, Southern University of Science and Technology, Shenzhen, Guangdong, 518055, P.R. China.

[3]Department of Mechanical and Energy Engineering, Southern University of Science and Technology, Shenzhen, Guangdong, 518055, P.R. China.

[#] These authors contributed equally to this work.

E-mail: geq@sustech.edu.cn, yangch@sustech.edu.cn

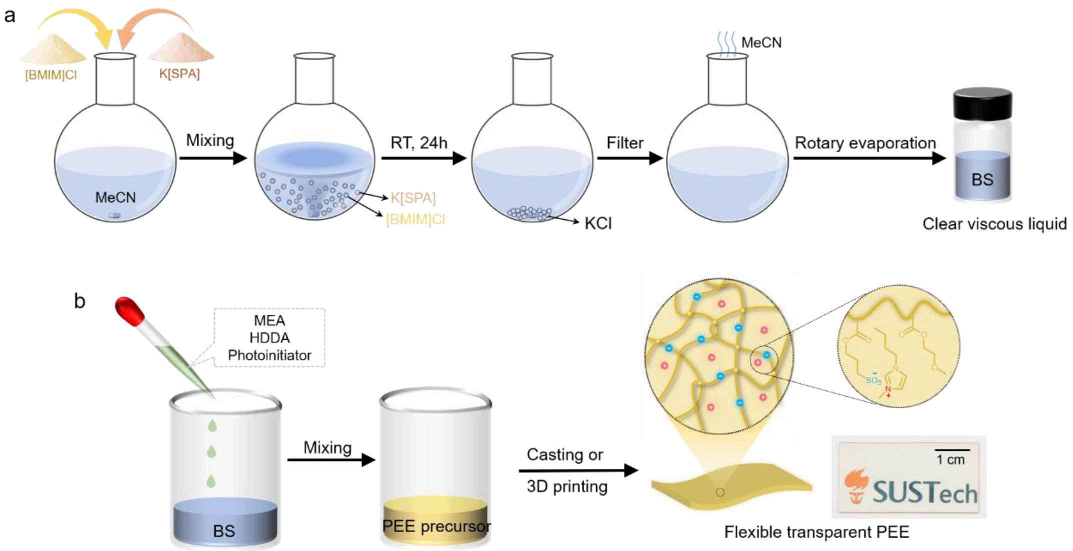

Figure S1. Synthesis of BS and p(BS-co-MEA). (a) [BMIM]Cl and K[SPA] are added into MeCN with the same molar mass. The mixture is stirred for 24 hours at room temperature, then the precipitate KCL is filtered out. After the rotary evaporation of the MeCN, a clear viscous liquid is obtained. (b) To synthesize p(BS-co-MEA), certain amounts of MEA, HDDA, and photo-initiator are added into BS to form the PEE precursor, which is then cast into a mold or 3D printed. After UV irradiation, the precursor cures, and a flexible and transparent PEE is obtained.

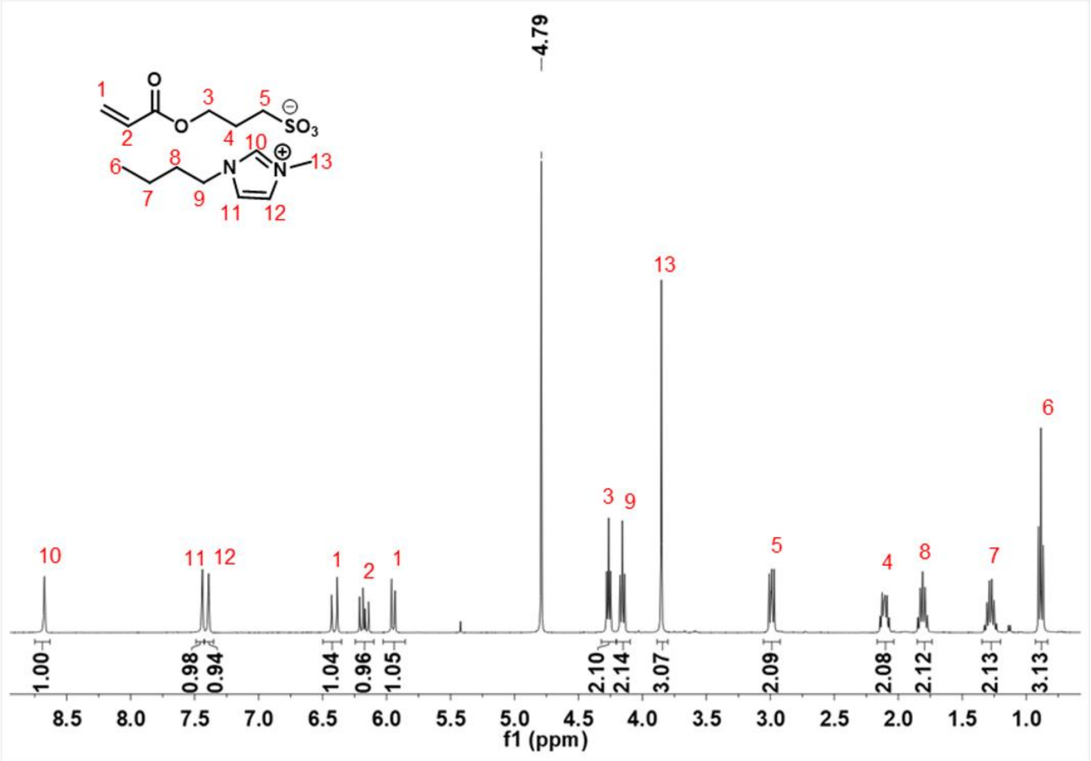

Figure S2. ^{1}H-NMR spectrum of BS: ^{1}H NMR (400 MHz, D_2O-d2) δ 8.75 (s, 1H), 7.49 (d, J = 19.1 Hz, 2H), 6.47 (d, J = 17.3 Hz, 1H), 6.24 (dd, J = 17.3, 10.5 Hz, 1H), 6.02 (d, J = 10.5 Hz, 1H), 4.32 (t, J = 6.3 Hz, 2H), 4.23 (t, J = 7.1 Hz, 2H), 3.92 (s, 3H), 3.08 - 3.00 (m, 2H), 2.22 - 2.11 (m, 2H), 1.92 - 1.83 (m, 2H), 1.42 - 1.28 (m, 2H), 0.95 (t, J = 7.4 Hz, 3H).

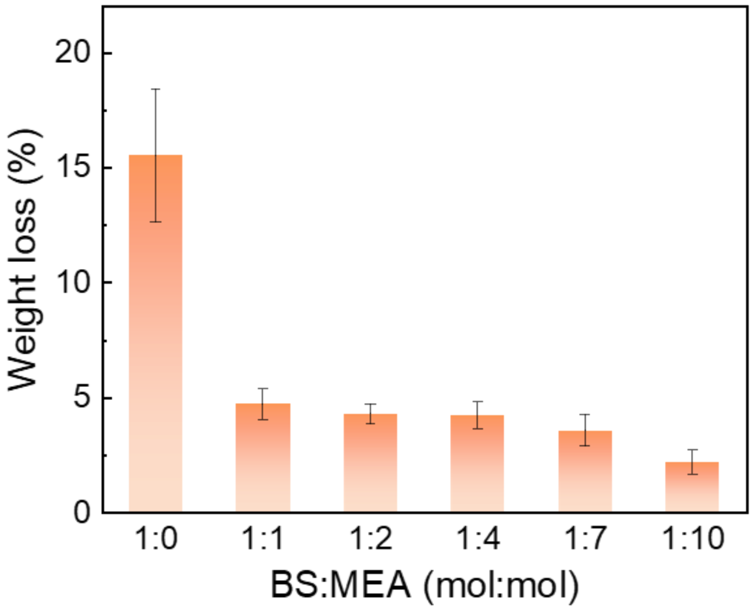

Figure S3. Weight loss of p(BS-co-MEA) after being stored in a 65 ^{o}C oven for 4 hours. The error bars represent standard deviations.

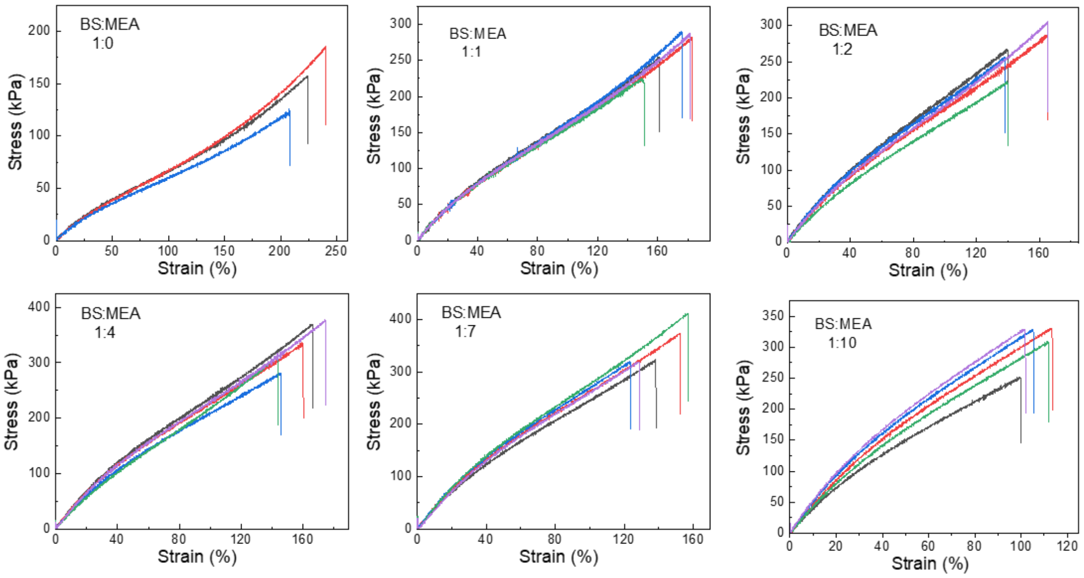

Figure S4. Uniaxial tensile stress-strain curves of p(BS-co-MEA) at various molar ratios of BS:MEA.

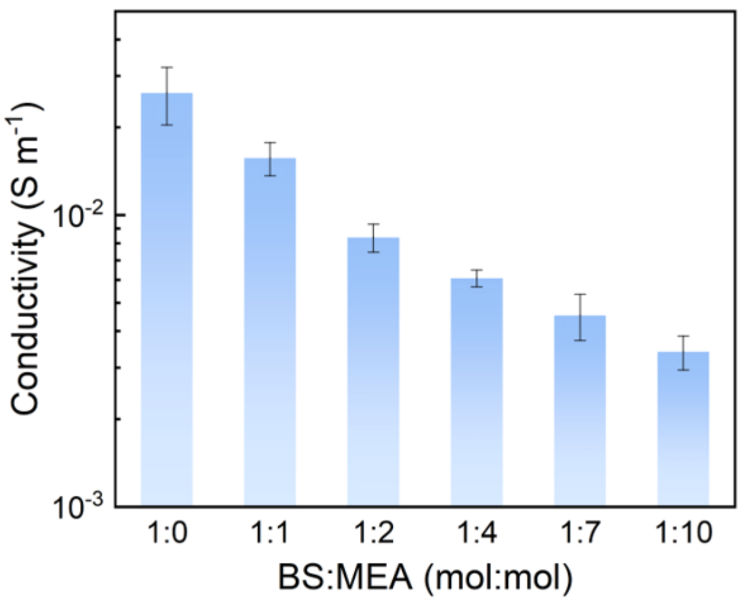

Figure S5. The variation of conductivity of p(BS-co-MEA) with the molar ratio of BS:MEA. The error bars represent standard deviations.

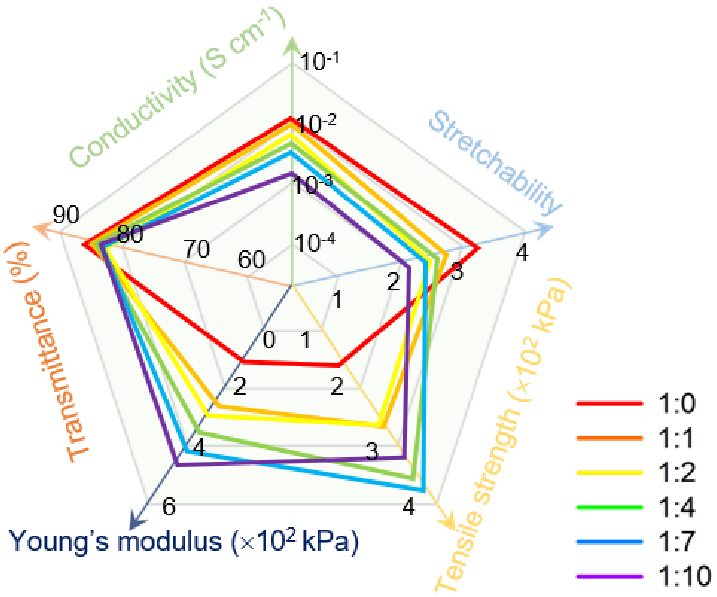

Figure S6. The comparison of various properties of p(BS-co-MEA) with different molar ratios of BS:MEA. The transmittance at 600 nm is used.

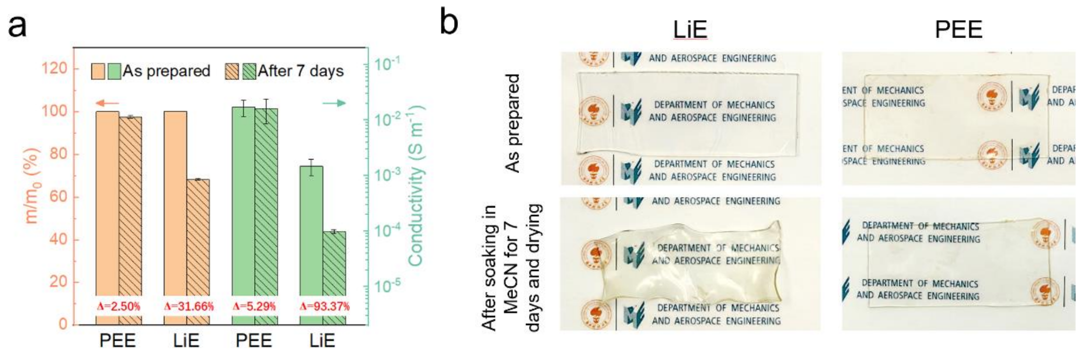

Figure S7. Stability of PEE and LiE in water. (a) The changes in weight and conductivity after soaking in water for 7 days and drying. (b) Images of LiE and PEE samples before and after the test. The error bars represent standard deviations.

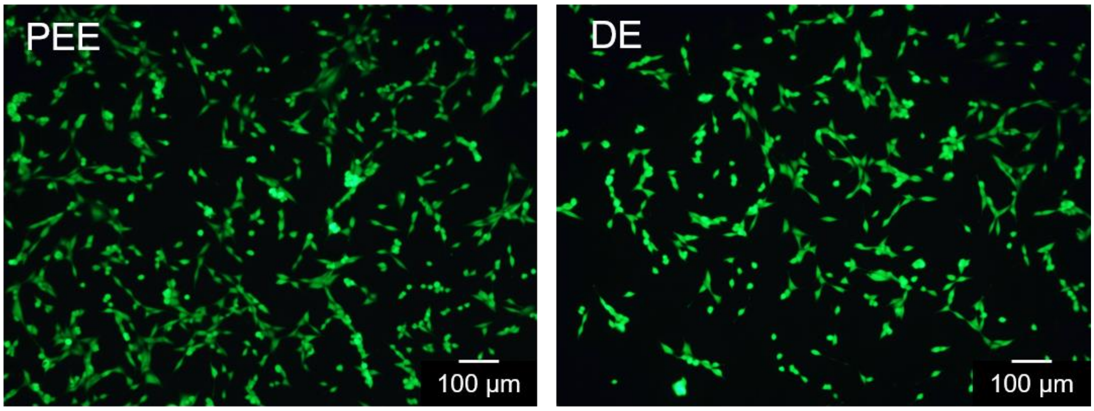

Figure S8. Live assay after 24 hours post seeding of NIH-3T3 cells cultured with PEE and DE slabs.

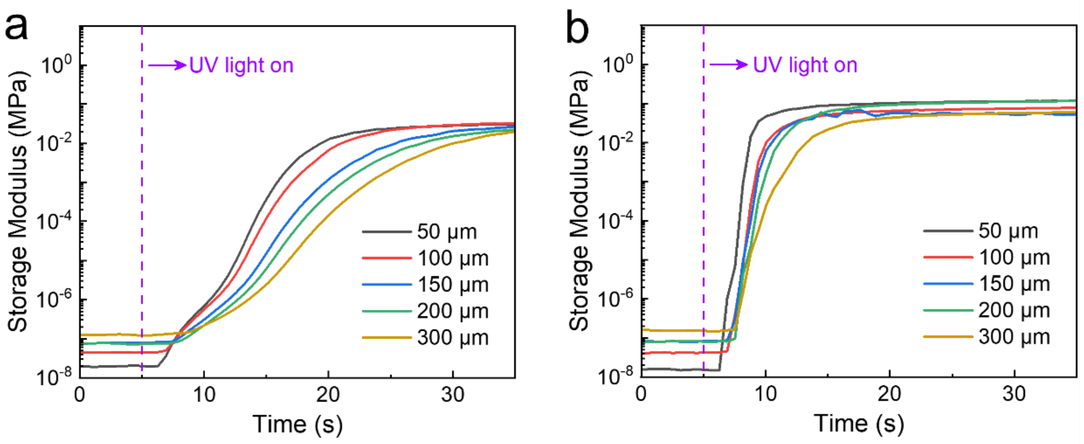

Figure S9. Photo-rheology of (a) PEE and (b) DE precursors with different layer thicknesses. The UV light of wavelength 405 nm and powder density 8.4 mW cm^{-2} was turned on at 5th s.

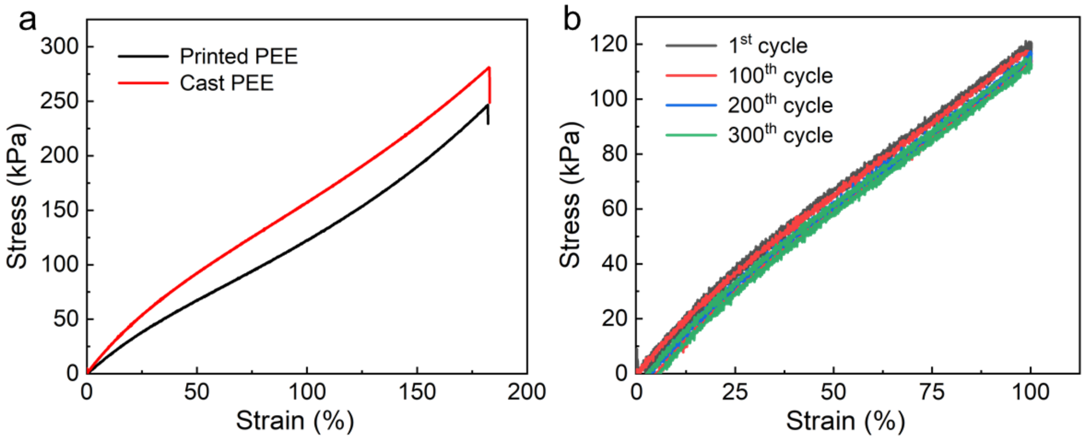

Figure S10. (a) Uniaxial stress-strain curves of printed PEE and cast PEE. The strains at break are 168 ± 18% for printed PEE and 170 ± 14% for cast PEE. The molar ratio of BS:MEA is 1:1. (b) Cyclic stress-strain curves with a maximum strain of 100% for 300 cycles. Excellent stability is manifested by the negligible degradation of mechanical properties.

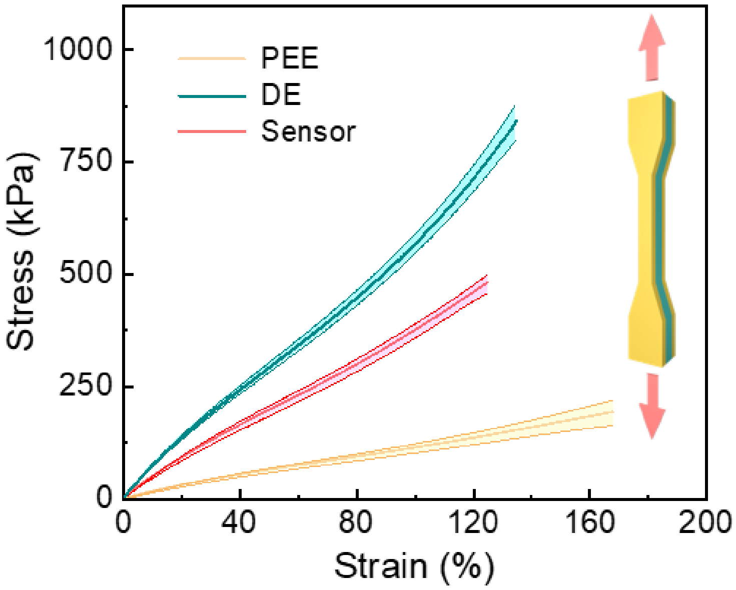

Figure S11. The comparison of the uniaxial tensile stress-strain curves of PEE, DE, and sensor. Each solid curve is averaged from at least three parallel samples and the shadows represent the standard deviations. The inset schematizes the uniaxial tension of a dumbbell-shaped sensor.

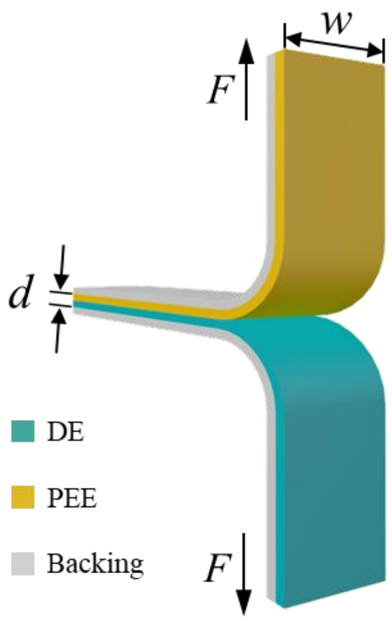

Figure S12. A schematic diagram of 180° peeling tests. The width of the sample is *w* and the total thickness of the sensor is *d*. The two arms of the sample are pulled by a pair of forces *F*. Two soft but inextensible backing layers are glued to the top and bottom surfaces to prevent elongation of the two arms so that, at steady state, the work done by the peel force is totally converted into fracture energy.

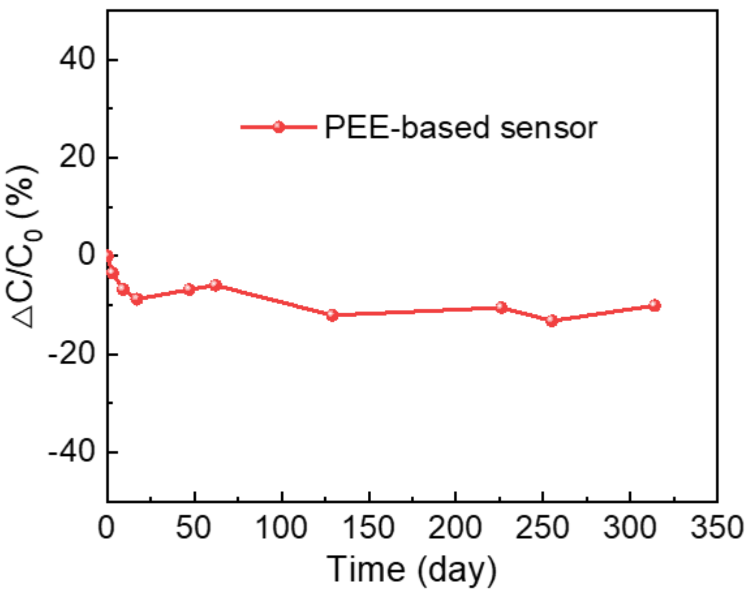

Figure S13. Durability test of PEE-based sensor. The variation of $\Delta C/C_0$ of the PEE-based sensor is ~10%, presumably caused by the fluctuations in temperature and humidity, after being stored in the ambient environment for 339 days.

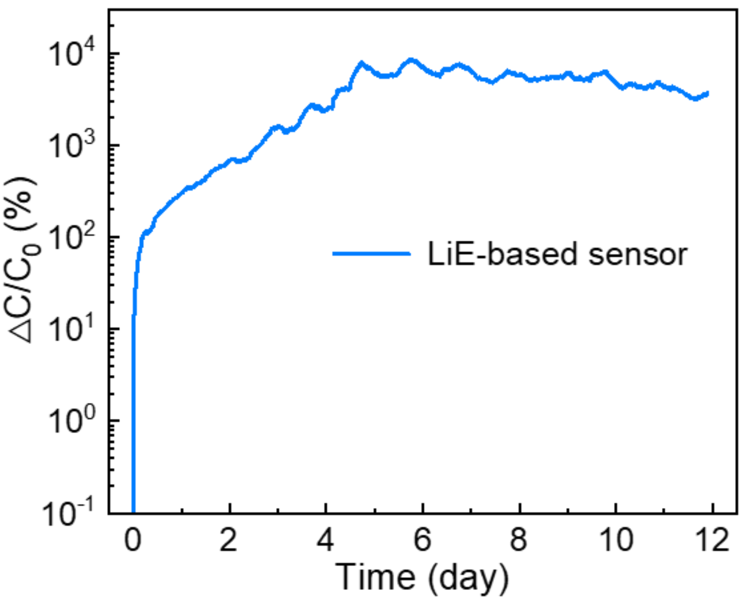

Figure S14. Durability test of LiE-based sensor. The capacitance of the sensor, with an initial value of ~30 pF, keeps increasing until plateauing at about ~1 nF after 4 days.

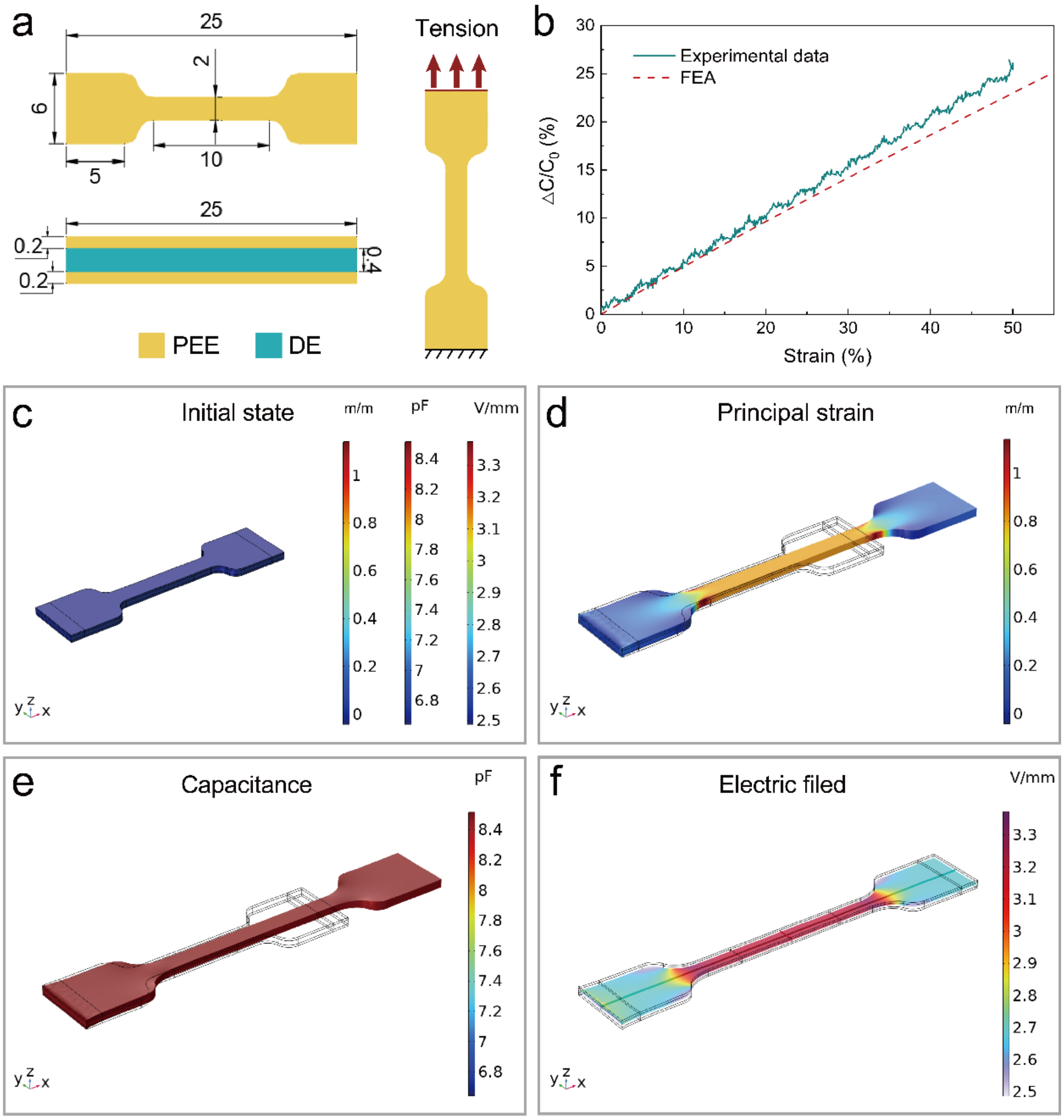

Figure S15. FEA results of the tensile sensor. (a) Geometries, boundary conditions, and loading conditions of the tensile sensor. (b) Variations of $\Delta C/C_0$ with tensile strain. Distributions of strain, capacitance, and electric field the sensor before (c) and after (d, e, f) deformation.

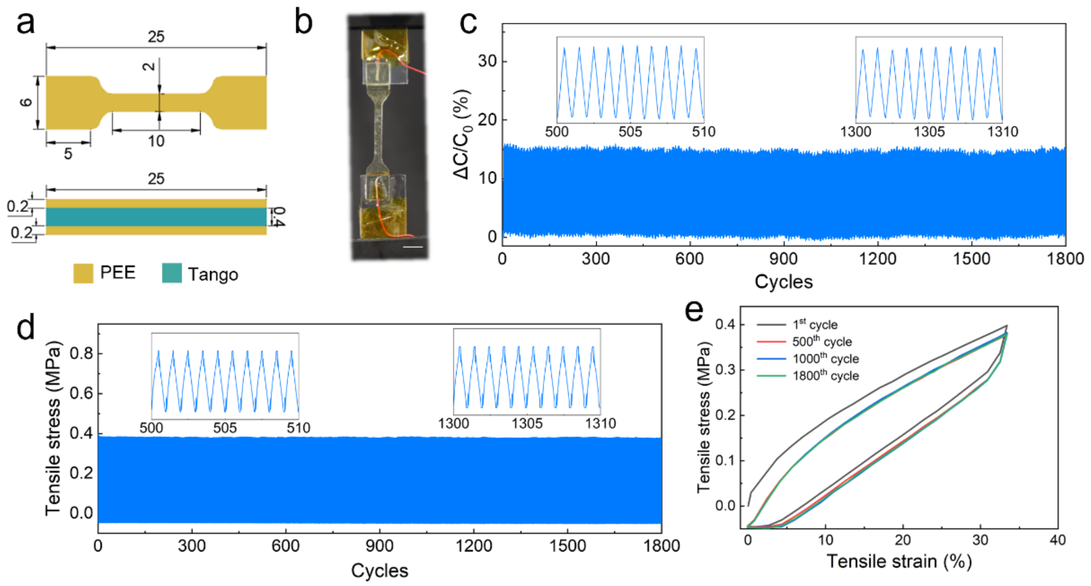

Figure S16. Cyclic tension of tensile sensor. (a) Schematics of the top view and side view of the dumbbell-shaped tensile sensor with relevant dimensions indicated. The unit in all schematics is in millimeters by default unless otherwise specified. (b) Photograph of the tensile sensor loaded to a mechanical testing machine and connected to an LCR meter. Scale bar: 5 mm. The variation of (c) $\Delta C/C_0$ and (d) tensile stress with the number of cycles. The maximum tensile strain is 33%. (e) Selected stress-strain curves during the cyclic test, implying excellent stability.

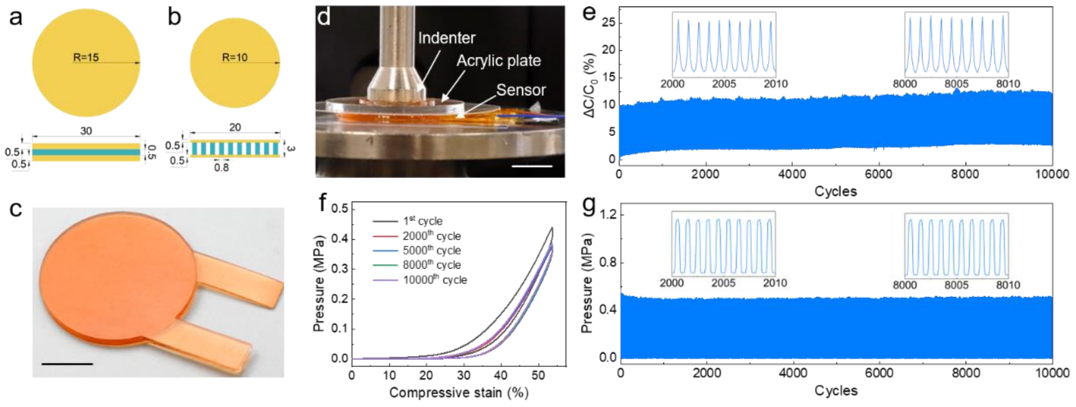

Figure S17. Cyclic compression of compressive sensor. (a) Schematics of the top view and side view of the working area of the compressive sensor with microstructure (a) and without microstructure (b), with relevant dimensions indicated. (c) Photograph of a compressive sensor without microstructure. Scale bar: 10 mm. (d) Photograph of the compressive sensor loaded to a mechanical testing machine and connected to an LCR meter. Scale bar: 10 mm. (e) Cyclic test of the compressive sensor without microstructure. (f) Selected stress-strain curves during the cyclic test, implying excellent stability. (g) Variation of pressure with the number of cycles. The maximum compressive displacement is 0.8 mm.

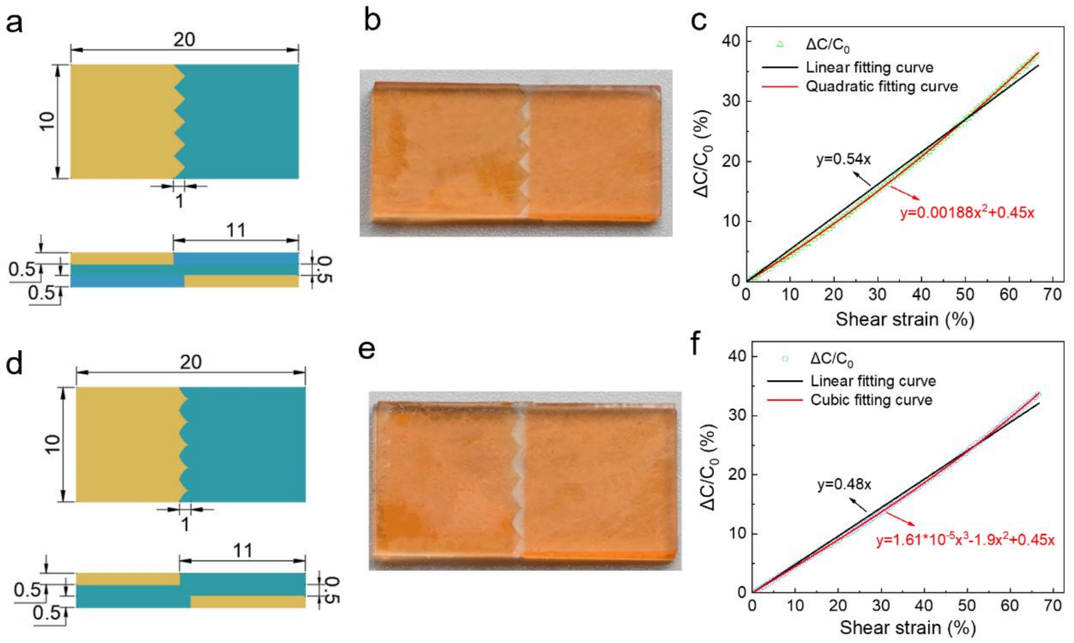

Figure S18. Shear sensors with zigzag and parabolic front lines. (a) Schematics of a sensor with a zigzag front line with relevant dimensions indicated. (b) Photograph of the sensor. Scale bar: 5 mm. (c) $\Delta C/C_0$ versus shear strain with two fitting curves, wherein the quadratic fitting is better than the linear fitting. (d) Schematics of a sensor with a parabolic front line with relevant dimensions indicated. (e) Photograph of the sensor. Scale bar: 5 mm. (f) $\Delta C/C_0$ versus shear strain with two fitting curves, wherein the cubic fitting is better than the linear fitting.

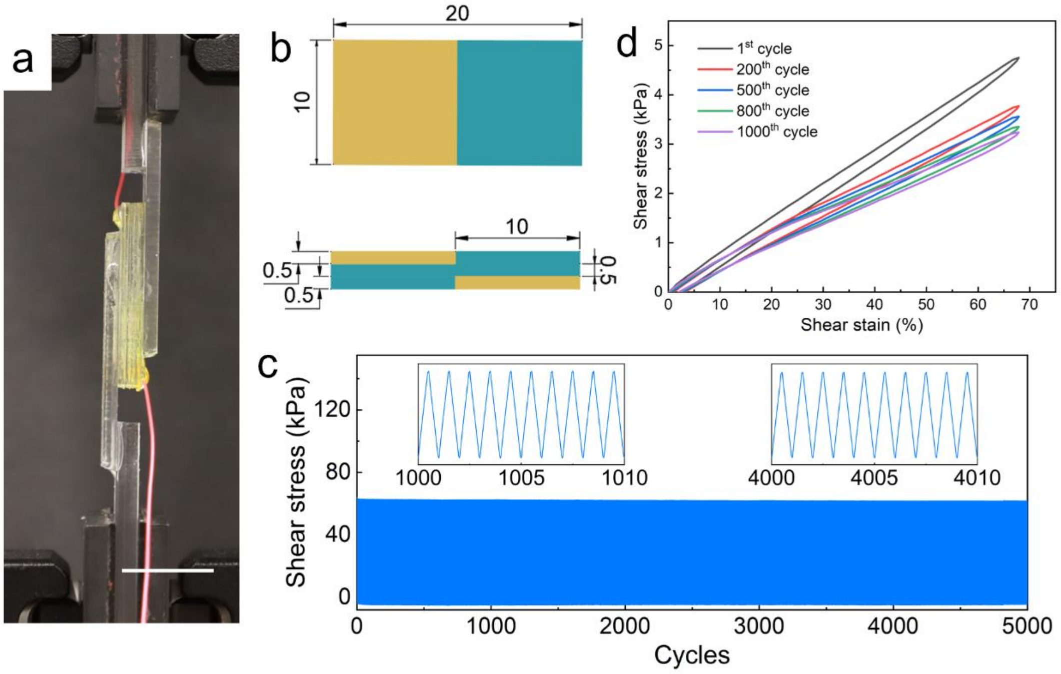

Figure S19. Cyclic shear of the shear sensor with a flat front line. (a) Photograph of the shear sensor loaded to a mechanical testing machine and connected to an LCR meter. Scale bar: 5 mm. (b) Schematics of the top view and side view of the sensor with relevant dimensions indicated. (c) Selected stress-strain curves during the cyclic test, implying excellent stability. (d) Shear stress varies with the number of cycles with a maximum shear displacement of 0.8 mm.

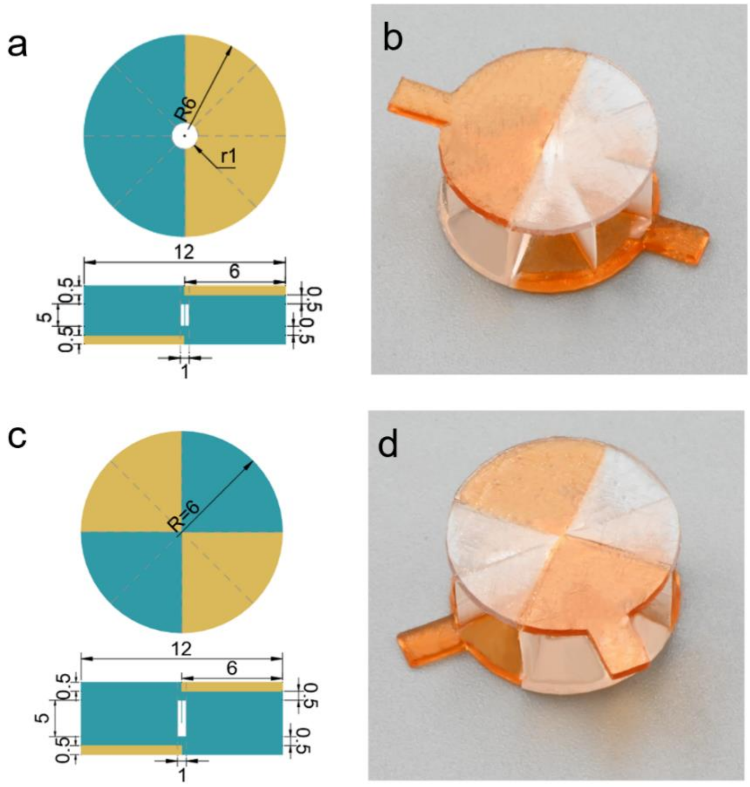

Figure S20. Torsional sensors of different designs. (a) Schematics of the top view and side view of a bisected torsional sensor with relevant dimensions indicated. (b) Photograph of the sensor. Scale bar: 5 mm. (c) Schematics of the top view and side view of a quartering torsional sensor with relevant dimensions indicated. (d) Photograph of the sensor. Scale bar: 5 mm.

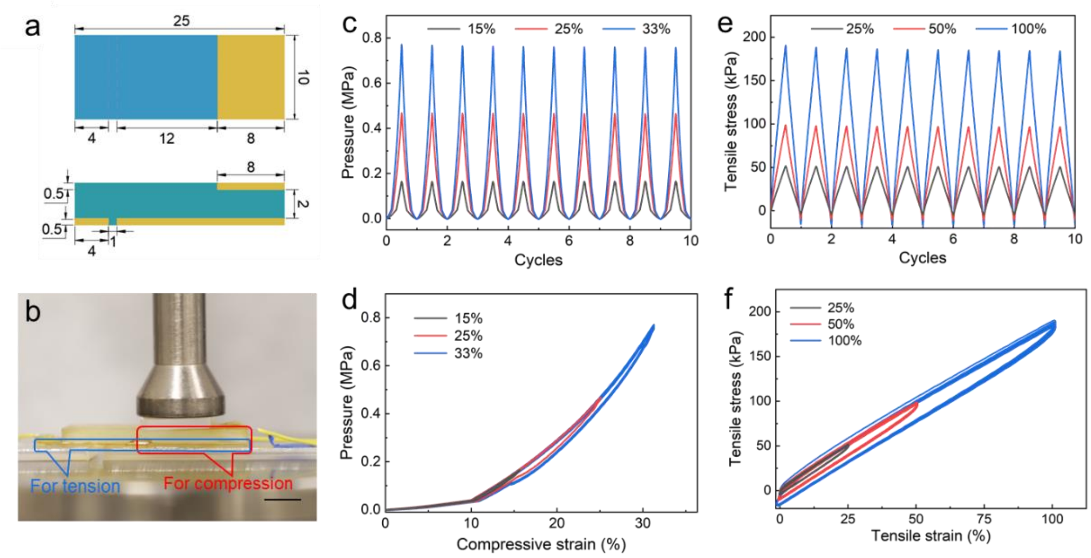

Figure S21. Integrated tensile and compressive ionotronic sensor. (a) Schematics of the top view and side view of the sensor with relevant dimensions indicated. (b) Photograph of a sensor loaded to a mechanical testing machine and connected to an LCR meter. Scale bar: 5mm. (c) Pressure varies with the number of cycles under cyclic compression with different compressive strains of 15%, 25%, and 33%. (d) Corresponding cyclic stress-strain curves. (e) Tensile stress varies with the number of cycles under cyclic tension with different tensile strains of 25%, 50%, and 100%. (f) Corresponding cyclic stress-strain curves.

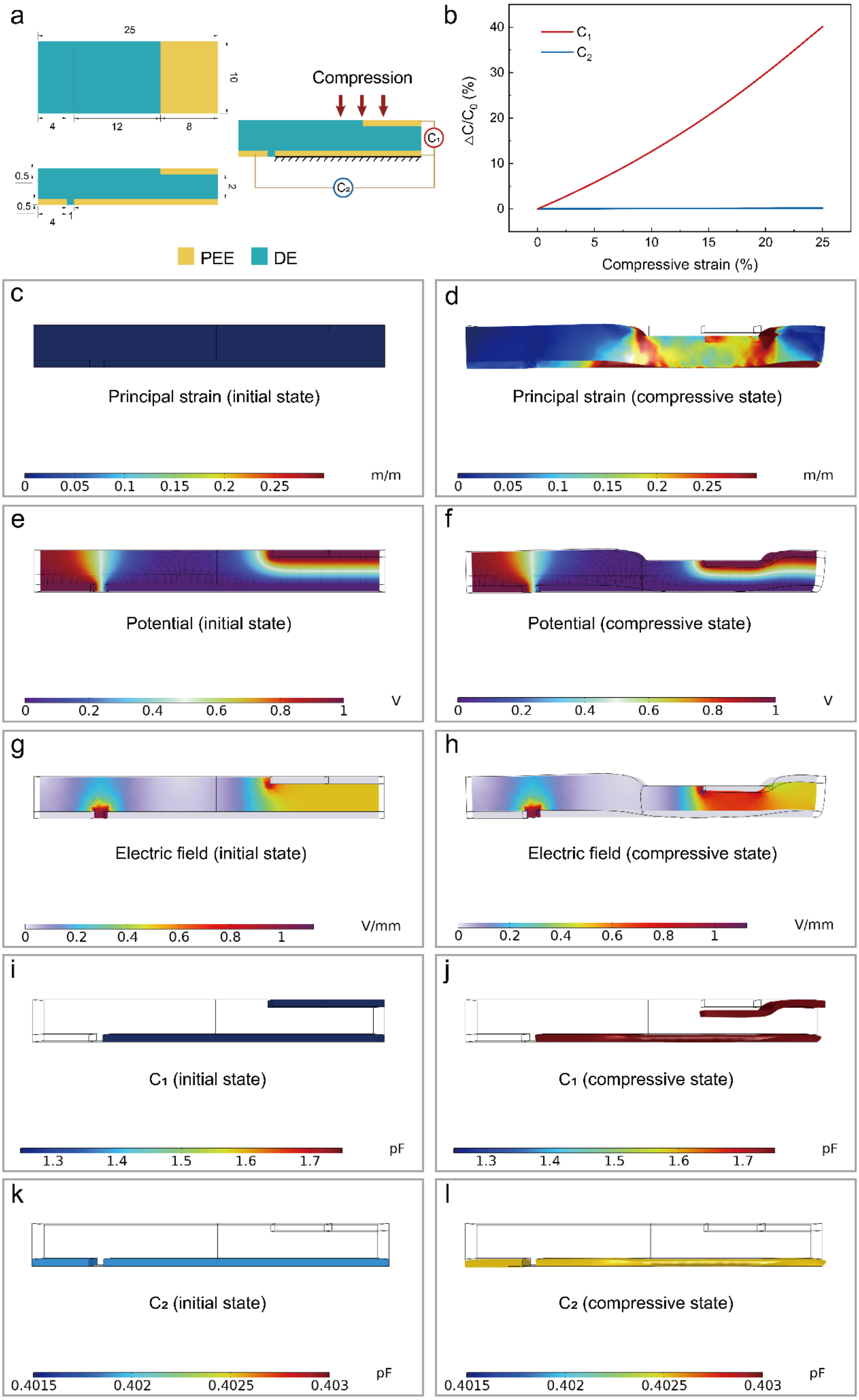
a
25
10
4
12
8
0.5
0.5
2
4
1
Compression
C₁
C₂
PEE
DE
b
C₁
C₂
ΔC/C₀ (%)
Compressive strain (%)
c
Principal strain (initial state)
m/m
d
Principal strain (compressive state)
m/m
e
Potential (initial state)
V
f
Potential (compressive state)
V
g
Electric field (initial state)
V/mm
h
Electric field (compressive state)
V/mm
i
C₁ (initial state)
pF
j
C₁ (compressive state)
pF
k
C₂ (initial state)
pF
l
C₂ (compressive state)
pF

Figure S22. FEA results of the integrated tensile and compressive sensor under compression. (a) Geometries, boundary conditions, and loading conditions of the integrated tensile and compressive sensor under compression. (b) $\Delta C/C_0$ varies with compressive strain for capacitance meters C_1 and C_2. Principal strain field distributions at undeformed state (c) and at a compressive strain of 25% (d). Potential field distributions at undeformed state (e) and at a compressive strain of 25% (f). Electric field distributions at undeformed state (g) and at a compressive strain of 25% (h). The capacitances of C_1 at undeformed state (i) and at a compressive strain of 25% (j). The capacitances of C_2 at undeformed state (k) and at a compressive strain of 25% (l).

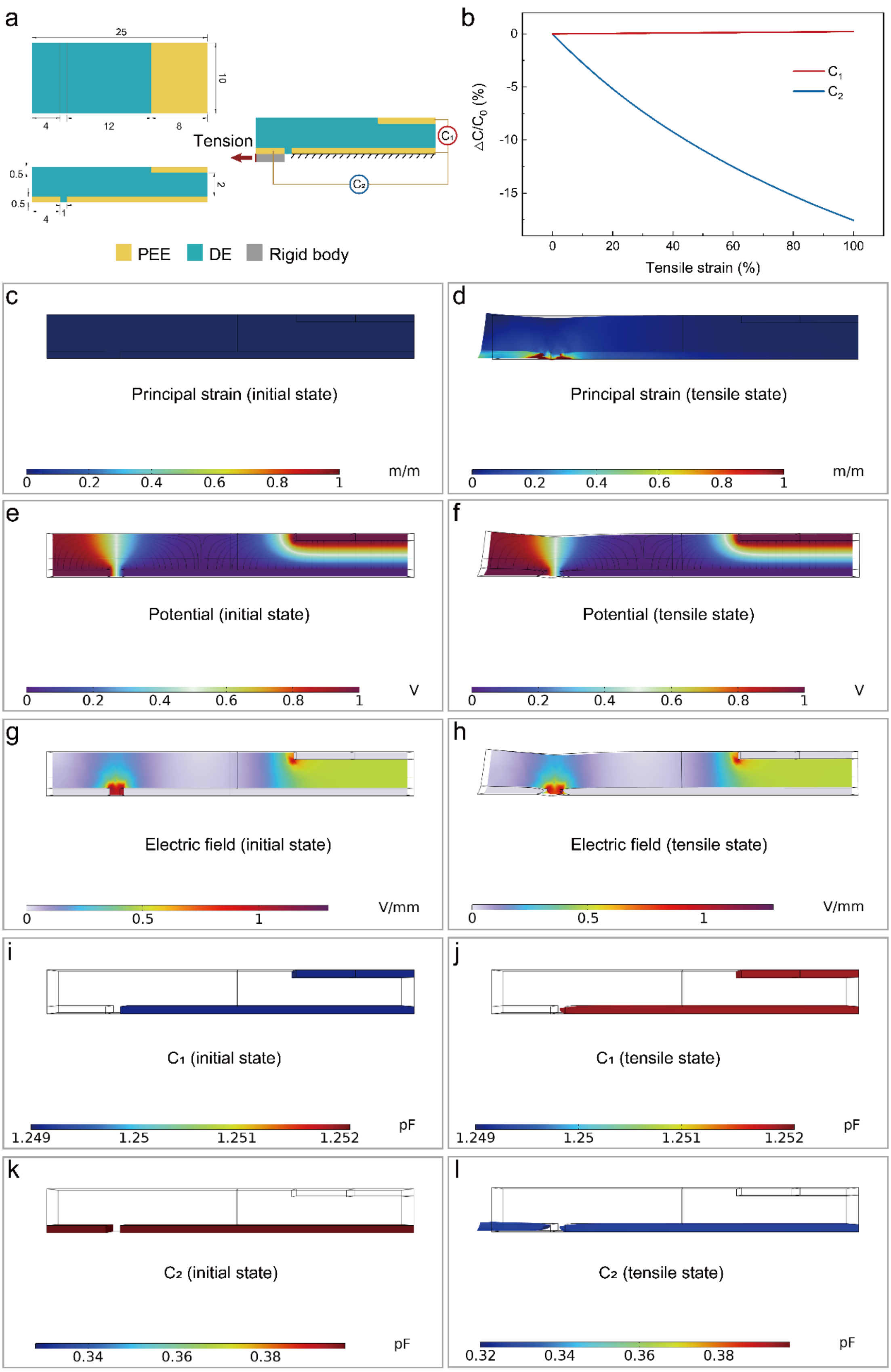

Figure S23. FEA results of the integrated tensile and compressive sensor under tension. (a) Geometries, boundary conditions, and loading conditions of the integrated tensile

and compressive sensor under tension. (b) $\Delta C/C_0$ varies with tensile strain for capacitance meters C_1 and C_2. Principal strain field distributions at undeformed state (c) and at a tensile strain of 100% (d). Potential field distributions at undeformed state (e) and at a tensile strain of 100% (f). Electric field distributions at undeformed state (g) and at a tensile strain of 100% (h). The capacitances of C_1 at undeformed state (i) and at a tensile strain of 100% (j). The capacitances of C_2 at undeformed state (k) and at a tensile strain of 100% (l).

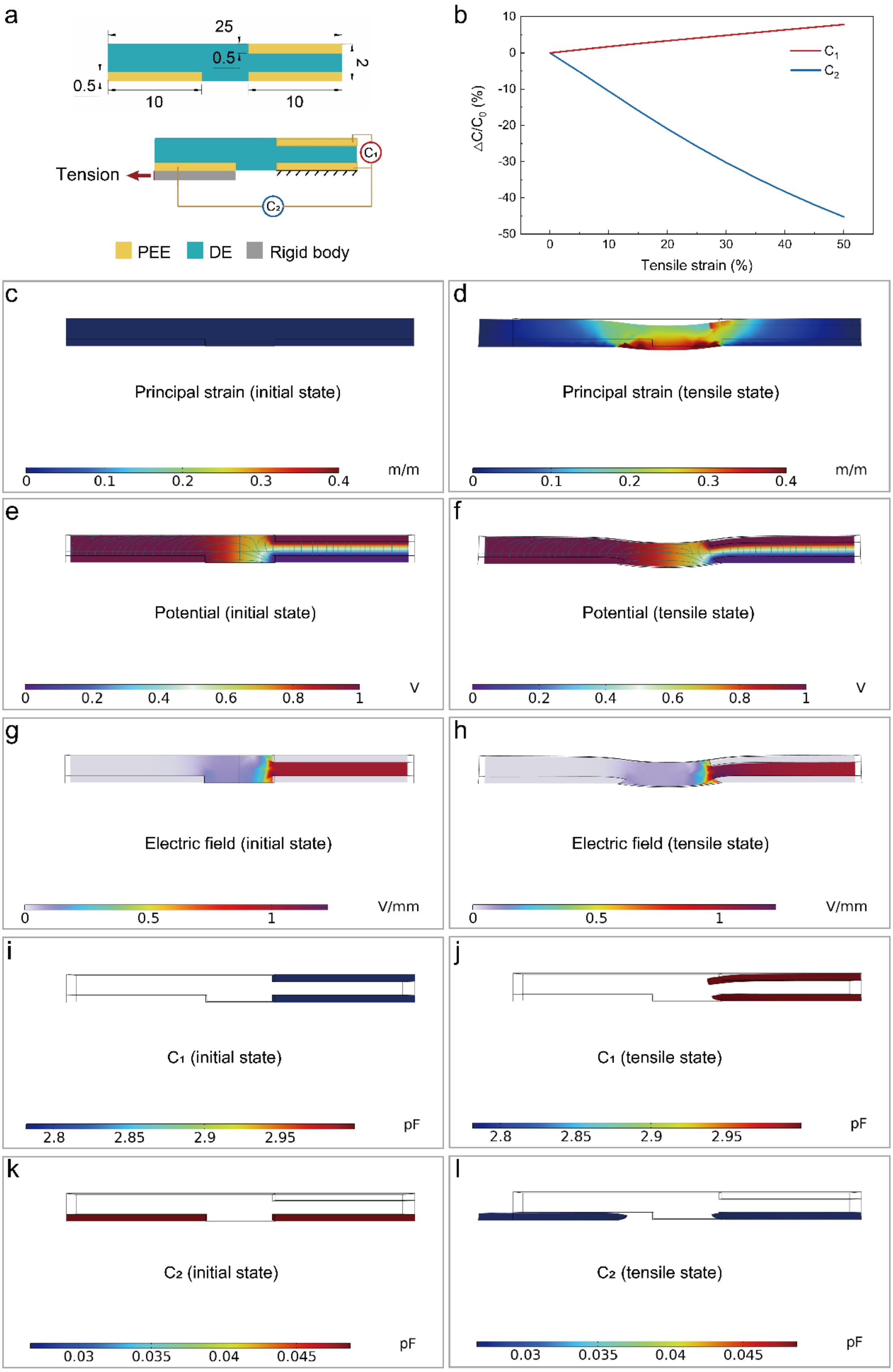

Figure S24. FEA results of a bad design of the integrated tensile and compressive sensor under tension. (a) Geometries, boundary conditions, and loading conditions of the

badly-designed integrated tensile and compressive sensor under tension. (b) $\Delta C/C_0$ varies with tensile strain for capacitance meters C_1 and C_2. Principal strain field distributions at undeformed state (c) and at a tensile strain of 50% (d). Potential field distributions at undeformed state (e) and at a tensile strain of 60% (f). Electric field distributions at undeformed state (g) and at a tensile strain of 50% (h). The capacitances of C_1 at undeformed state (i) and at a tensile strain of 50% (j). The capacitances of C_2 at undeformed state (k) and at a tensile strain of 50% (l).

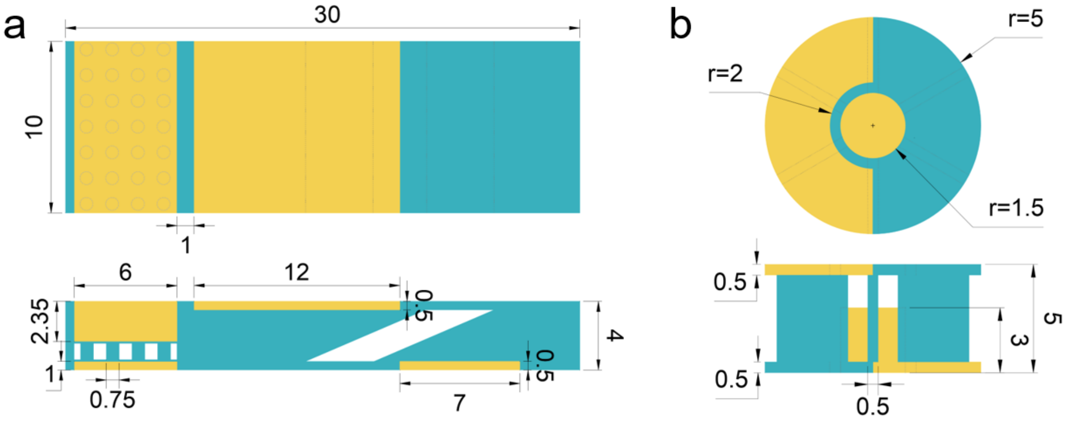

Figure S25. Schematics of the top view and side view of (a) integrated compressive and shear sensor and (b) integrated compressive and torsional sensor, with relevant dimensions indicated.

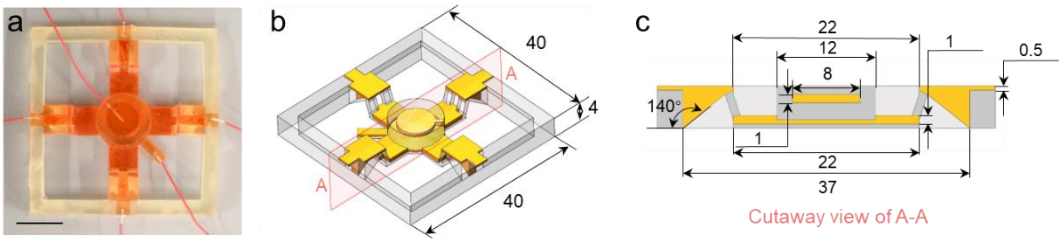

Figure S26. Remote-control sensor. (a) Photograph of the remote-control sensor with copper wires connected. Scale bar: 10 mm. (b) Schematic of the remote-control sensor with relevant dimensions indicated. (c) Schematic of the cutaway view with relevant dimensions indicated. The unit is mm.

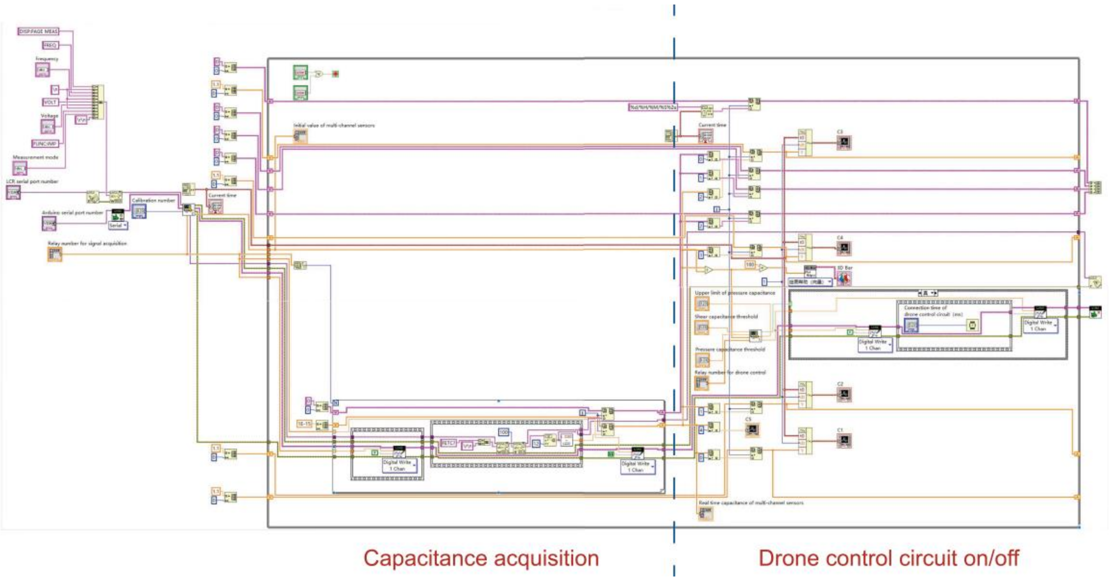

Figure S27. The LabVIEW program for the remote-control system. The program runs from left to right. First, set the parameters for capacitance measurement outside the loop structure and calibrate the initial values of each capacitance channel. Then, assign one channel of the relays to each capacitor after entering the loop structure. When measurement starts, close the circuit of one channel at a time meanwhile keeping other channels open, measure the capacitance of the closed channel, and then disconnect the channel. Repeat the process for five channels. After that, enter the drone control circuit and compare the five capacitance values with the initial values. Finally, perform a closed-circuit operation to generate an action command to the drone based on the corresponding relay channel.

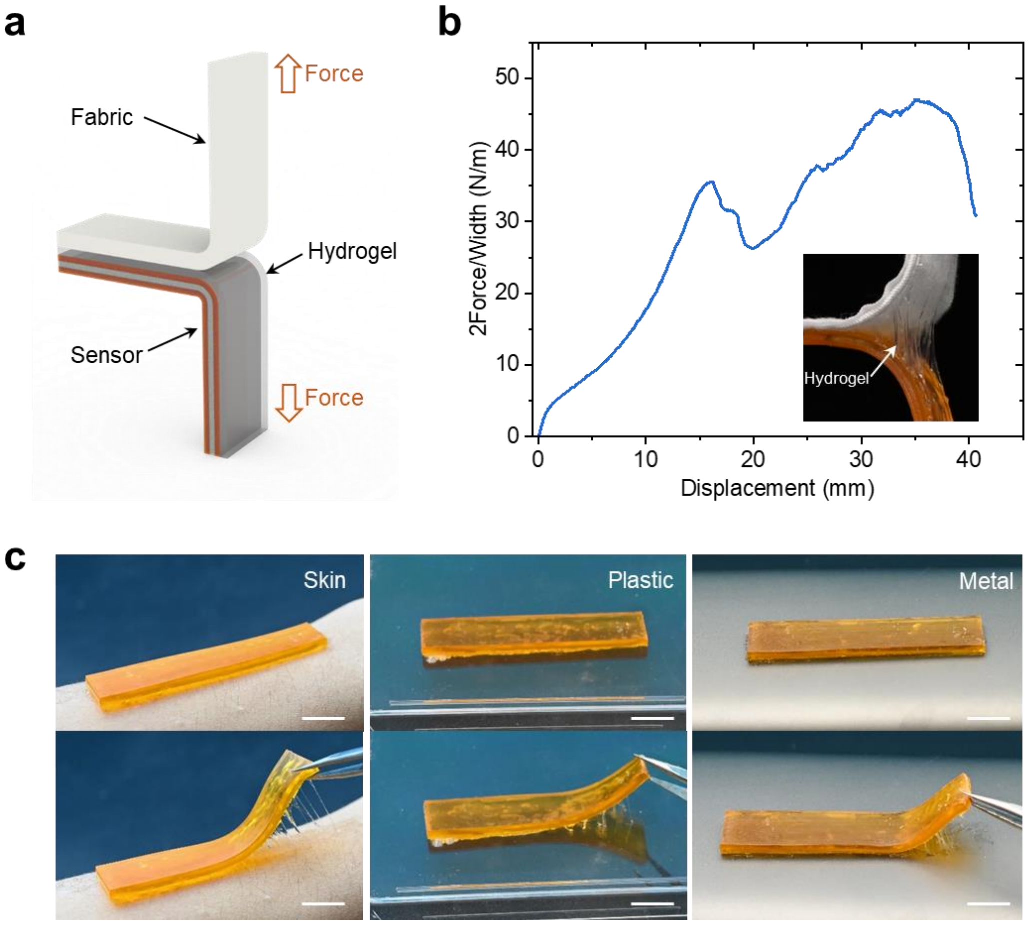

Figure S28. A schematic diagram (a) and test data (b) of the 180° peeling test for 3D printed sensor and fabric with a hydrogel as an adhesive layer. (c) Photographs of a 3D-printed sensor adhere to skin, plastic, and metal (aluminum alloy). Scale bar: 10mm.

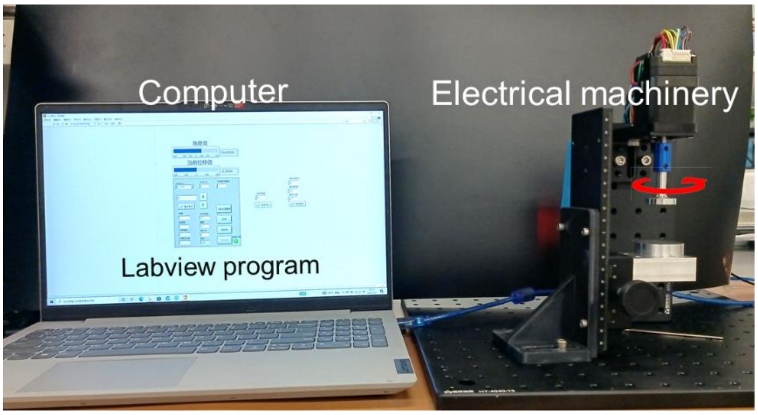

Figure S29. Photograph of the self-built LabVIEW-controlled torsional loading system.

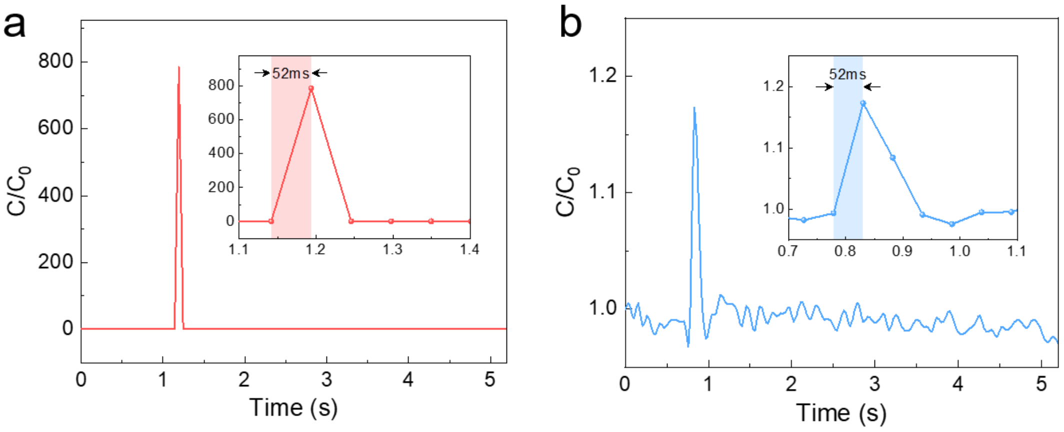

Figure S30. Response time tests of (a) shear sensor and (b) compressive sensor of the remote-control unit.

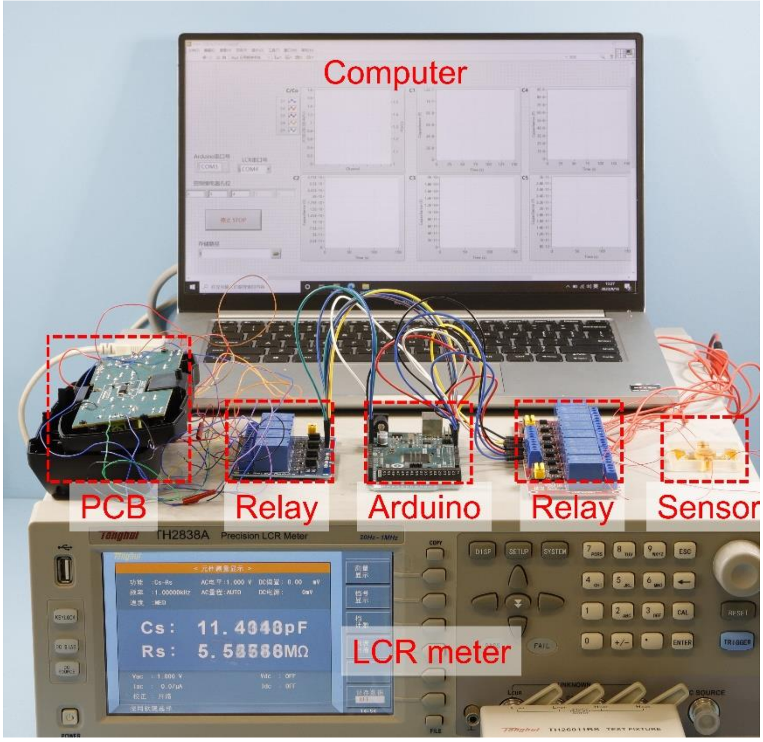

Figure S31. Photograph of the remote-control system for a drone with the 3D printed sensor as the input port.

Table S1. The comparison of various ionotronic sensors

	Ionic conductor	Tension	Compression	Shear	Torsion	Combined deformation
ref[9]	Hydrogel	√	√	×	×	×
ref[S1]	Hydrogel	√	√	×	×	×
ref[S2]	Hydrogel	√	×	×	×	×
ref[17]	Hydrogel	√	√	×	×	×
ref[S3]	Hydrogel	√	×	×	×	×
ref[S4]	Hydrogel	√	√	×	×	×
ref[S5]	Hydrogel	√	×	×	×	×
ref[S6]	Ionogel	√	×	×	×	×
ref[S7]	Ionogel	√	×	×	×	×
ref[S8]	Ionogel	×	√	×	×	×
ref[S9]	Ionogel	√	×	×	×	×
ref[S10]	Ionogel	√	×	×	×	×
ref[S11]	Ionogel	√	√	×	×	×
ref[S12]	Ionogel	√	×	×	×	×
ref[S13]	Ionogel	×	×	√	×	×
ref[S14]	Ionogel	√	×	×	×	×
ref[31]	ICE[a]	√	√	×	×	×
ref[S15]	ICE	×	√	×	×	×
ref[S16]	ICE	√	×	×	×	×
ref[S17]	ICE	√	×	×	×	×
ref[S18]	PEE	√	×	×	×	×
This work	PEE	√	√	√	√	√

[a] ICE denotes the ionically conductive elastomer made by dissolving salt into an elastomer matrix.

Supplementary References

[S1] C. Larson, B. Peele, S. Li, S. Robinson, M. Totaro, L. Beccai, B. Mazzolai, R. Shepherd, *Science* **2016**, 351, 1071.

[S2] G. Ge, Y. Lu, X. Qu, W. Zhao, Y. Ren, W. Wang, Q. Wang, W. Huang, X. Dong, *Acs Nano* **2020**, 14, 218.

[S3] X. Yuan, P. Wu, Q. Gao, J. Xu, B. Guo, Y. He, *Materials Horizons* **2022**, 9.

[S4] Z. Huang, Z. Deng, X. Liu, T. Huang, Y. Hu, Y. Chen, Y. Liu, Z.-H. Guo, K. Yue, *Chemical Engineering Journal* **2022**, 449.

[S5] R. Xu, M. She, J. Liu, S. Zhao, H. Liu, L. Qu, M. Tian, *Advanced Fiber Materials* **2022**, DOI: 10.1007/s42765-022-00186-z.

[S6] Y. Ren, J. Guo, Z. Liu, Z. Sun, Y. Wu, L. Liu, F. Yang, *Science Advances* **2019**, 5.

[S7] Y. M. Kim, H. C. Moon, *Advanced Functional Materials* **2020**, 30.

[S8] K. Keum, J. Eom, J. H. Lee, J. S. Heo, S. K. Park, Y.-H. Kim, *Nano Energy* **2021**, 79.

[S9] Z. Yu, P. Wu, *Advanced Materials* **2021**, 33.

[S10] B. Yiming, X. Guo, N. Ali, N. Zhang, X. Zhang, Z. Han, Y. Lu, Z. Wu, X. Fan, Z. Jia, S. Qu, *Advanced Functional Materials* **2021**, 31.

[S11] L. Xu, Z. Huang, Z. Deng, Z. Du, T. L. Sun, Z.-H. Guo, K. Yue, *Advanced Materials* **2021**, 33.

[S12] M. Ma, Y. Shang, H. Shen, W. Li, Q. Wang, *Chemical Engineering Journal* **2021**, 420.

[S13] W. Suh, C. Park, J. Oh, S. Moon, S. Choi, Y. S. Kim, U. Jeong, *Advanced Materials Technologies* **2022**, 7.

[S14] W. Qiu, G. Chen, H. Zhu, Q. Zhang, S. Zhu, *Chemical Engineering Journal* **2022**, 434.

[S15] Q. Li, Z. Liu, S. Zheng, W. Li, Y. Ren, L. Li, F. Yan, *Acs Applied Materials & Interfaces* **2022**, 14, 26068.

[S16] B. Zhang, Q. Feng, H. Song, X. Zhang, C. Zhang, T. Liu, *Acs Applied Materials & Interfaces* **2022**, 14, 8404.

[S17] R. Borayek, F. Foroughi, X. Xin, A. M. Mohamed, M. M. Abdelrahman, M. Zedan, D. Zhang, J. Ding, *Acs Applied Materials & Interfaces* **2022**, 14, 11727.

[S18] X. Ming, C. Zhang, J. Cai, H. Zhu, Q. Zhang, S. Zhu, *Acs Applied Materials & Interfaces* **2021**, 13, 31102.
