# Supplementary material for: Polyelectrolyte elastomer-based ionotronic sensors with multi-mode sensing capabilities via multi-material 3D printing
Source: Nat Commun. 2023 Aug 10;14:4853. doi: 10.1038/s41467-023-40583-5 (PMC10415297; doi:10.1038/s41467-023-40583-5)
Supplement: Supplementary file 3 — Description of Additional Supplementary Files [file 41467_2023_40583_MOESM3_ESM.pdf]

Description of Additional Supplementary Files

Supplementary Movie 1: Performances of integrated compressive and shear sensor

Description: Two acrylic plates are glued to the top and bottom surfaces of the sensor and the bottom plate was fixed to a substrate. Compressive load and shear load are applied by pressing and pushing the upper acrylic plate, from the top and the left respectively, with a finger. The capacitance changes in the two sensors are alternately measured by a relay and an LCR meter, and the real-time signals are displayed on the screen of a laptop.

Supplementary Movie 2: Characterization of the remote-control unit.

Description: The five sensors of the remote-control unit are connected to an external measuring circuit and their real-time capacitances and normalized capacitances are displayed. When a finger applies force to activate a sensor, the capacitance of that sensor increases drastically, which is highlighted by a red box. The capacitance decreases after the force is removed.

Supplementary Movie 3: Remote-control system for a drone.

Description: The remote-control system contains a remote-control unit, two relays, an LCR meter, a LabVIEW controlling program, and an Arduino board. The remote-control unit is worn on the hand back. When the sensors of C_1, C_2, C_4, C_5 + C_2, C_4, and C_3 are activated sequentially, the drone flies according to the corresponding orders of “up”, “left”, “right”, “flip to the left”, “right”, and “down”.
